# A quantitative and mechanistic model for monoclonal antibody glycosylation as a function of nutrient availability during cell culture

**DOI:** 10.1186/1753-6561-7-S6-O10

**Published:** 2013-12-04

**Authors:** Ioscani Jiménez del Val, Antony Constantinou, Anne Dell, Stuart Haslam, Karen M Polizzi, Cleo Kontoravdi

**Affiliations:** 1 Centre for Process Systems Engineering, Department of Chemical Engineering, Imperial College London, South Kensington Campus, London, SW7 2AZ, UK; 2 Department of Life Sciences, Imperial College London, South Kensington Campus, London, SW7 2AZ, UK; 3 Centre for Synthetic Biology and Innovation, Imperial College London, South Kensington Campus, London, SW7 2AZ, UK

## Introduction

Monoclonal antibodies (mAbs) are currently the highest-selling products of the biopharmaceutical industry, having had global sales of over $45 billion in 2012 [1]. All commercially-available mAbs contain a consensus N-linked glycosylation site on each of the Cγ2 domains of their constant fragment (Fc). The monosaccharide composition and distribution of these N-linked carbohydrates (glycans) has been widely reported to directly impact the safety and efficacy of mAbs when administered to patients. Many studies have also shown that manufacturing bioprocess conditions (e.g. nutrient availability, metabolite accumulation, dissolved oxygen, pH, temperature and stirring speed) directly influence the composition and distribution of N-linked glycans bound to mAbs and other recombinant proteins. Given this tight interconnection between manufacturing process conditions, product quality and ensuing safety and therapeutic efficacy, mAbs and their glycosylation present a clear opportunity where process development can be guided by quality by design (QbD) principles.

QbD is a conceptual framework that aims to build quality into drug products at every stage of process development. Specifically, implementation of QbD to pharmaceutical process development requires identifying critical quality attributes (CQAs) that define the drug's safety and therapeutic efficacy. QbD then uses all available information on the mechanisms that quantitatively relate process inputs with product quality to control the manufacturing process so that product CQAs are maintained and end-product quality is ensured. Within the QbD context, composition and distribution of the glycans present on the Fc of mAbs is defined as a CQA, and thus, the processes employed in their manufacture must be controlled so that their glycan distribution ensures the required safety and efficacy profiles. Under this perspective, we have defined a mathematical model that mechanistically and quantitatively describes mAb Fc glycosylation as a function of nutrient availability during cell culture. Such a model aims to be used for bioprocess design, control and optimisation, thus facilitating the manufacture of mAbs with built-in glycosylation-associated quality under the QbD scope.

## Materials and methods

The mathematical model consists of three distinct modular elements which have been connected to achieve a mechanistic description of mAb glycosylation as a function of nutrient availability. The first element corresponds to cell culture dynamics and uses modified Monod kinetics to describe the growth and death of cells as a function of glucose and glutamine availability. This element also describes accumulation of metabolites (lactate and ammonia) and mAb synthesis throughout cell culture.

**Figure 1:**
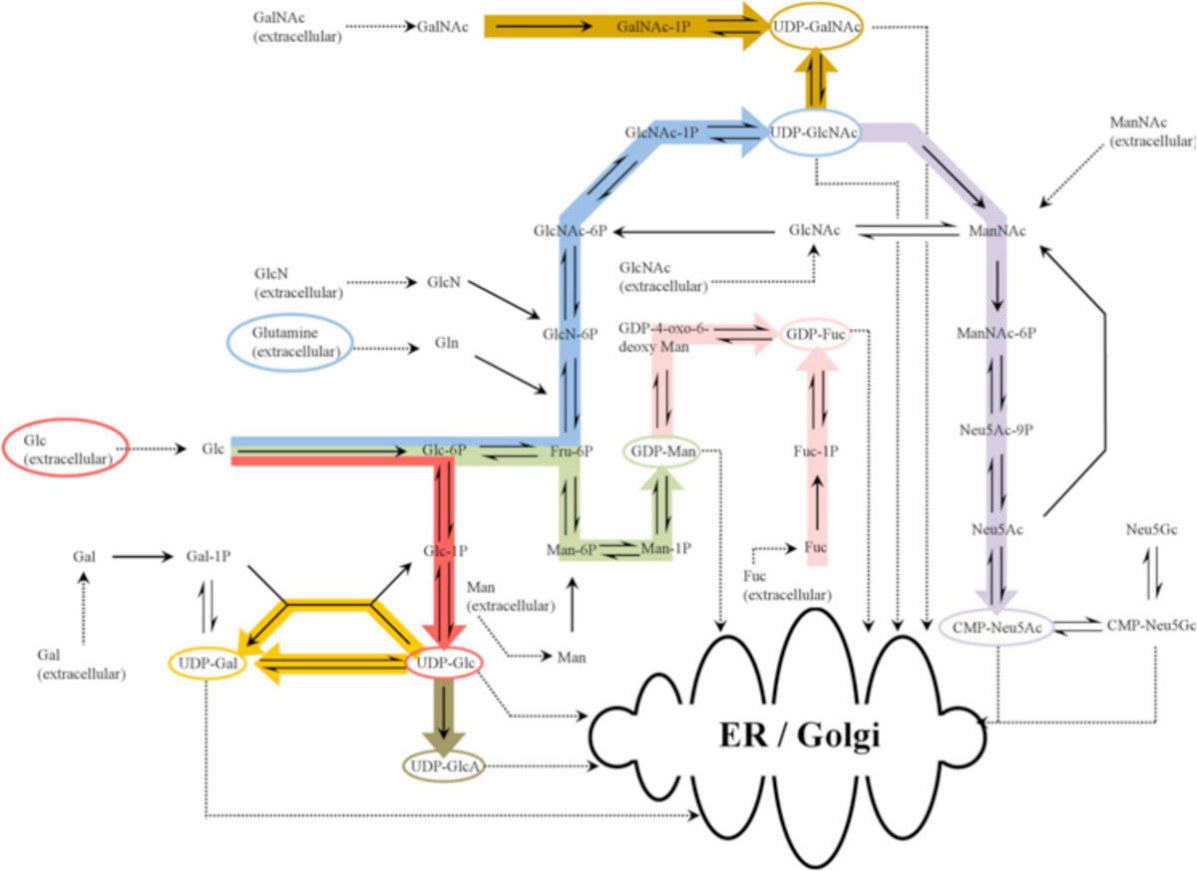
**Nucleotide sugar metabolic network**.

The second element describes the intracellular dynamics of nucleotide sugar (NS) metabolism. NSs are the substrates required for protein glycosylation and are synthesised via the amino sugar and nucleotide sugar metabolic pathway using glucose and glutamine as primary substrates [2]. The full metabolic pathway has been heuristically reduced to 8 reactions by collapsing sequential reactions along each distinct branch of the pathway into a single one, as shown with the coloured arrows in Figure 1. This module is linked with the cell culture dynamics one by equations that define intracellular glucose and glutamine accumulation as a function of their availability in the extracellular environment.

The pathway shows the synthesis of uridine diphosphate N-acetylglucosamine (UDP-GlcNAc), uridine diphosphate N-acetylgalactosamine (UDP-GalNAc), uridine diphosphate glucose (UDP-Glc), uridine diphosphate galactose (UDP-Gal), guanosine diphosphate mannose (GDP-Man), guanosine diphosphate fucose (GDP-Fuc), cytosine monophosphate N-acetylneuraminic acid (CMP-Neu5Ac) and uridine diphosphate glucoronic acid (UDP-GlcA) using glucose (Glc) and glutamine as substrates. The coloured arrows represent the reduced scheme where sequential reactions have been collapsed into a single one (e.g. the blue arrow describes a single reaction that produces UDP-GlcNAc using glucose and glutamine as substrates). The remaining arrows represent the synthesis of the other NSs using glucose and glutamine or other NSs as substrates.

The third element describes mAb Fc glycosylation as a function of mAb specific productivity and NS availability. This element approximates the Golgi apparatus to a plug-flow reactor and considers the transport of NSs from the cytosol, where they are synthesised, into the Golgi, where they are consumed in glycosylation reactions [3]. As inputs, this element requires intracellular NS availability and mAb specific productivity, and is thus coupled to the other two modules. All model simulation was performed with gPROMS v. 3.4.0 [4].

Experimentally, murine hybridoma cells (CRL-1606, ATCC) were cultured and typical data was collected (viable cell density, extracellular glucose, glutamine, lactate, ammonia and mAb titre). In addition, the intracellular pools of NSs were extracted using perchloric acid and quantified using a high performance anion exchange chromatographic method that allows for quantification of 8 NSs and 8 nucleotides in under 30 minutes [5]. Finally, the mAb glycan profiles were obtained using MALDI mass spectrometry.

The obtained experimental data was then used to estimate the unknown parameters of the model. Estimation was performed with the maximum likelihood formulation available in gPROMS v. 3.4.0, where the values for uncertain physical parameters are obtained to maximise the probability that the model will predict values from experimental measurements [4].

## Results

Time-courses for all data were produced, including intracellular profiles for six NSs (GDP-Man, GDP-Fuc, UDP-Glc, UDP-Gal, UDP-GlcNAc and CMP-Neu5Ac). This, along with data on cell culture dynamics and mAb Fc glycosylation were used to estimate the unknown parameters of the model as described previously. With the estimated parameters, the mathematical model was found to reproduce cell culture dynamics, intracellular NS pools and terminal mAb Fc glycan distributions accurately.

With the obtained parameters, a case study for glutamine depletion was simulated. This study showed that under glutamine deprivation, intracellular availability of UDP-GlcNAc decreases to a point where mAbs with high-mannose (Man5) glycan structures begin accumulating in the extracellular environment, a phenomenon that is consistent with previous observations [6].

## Conclusions

We have shown the construction of a mathematical model which mechanistically and quantitatively describes mAb Fc glycosylation as a function of nutrient availability during cell culture. In addition, experimental methods have been developed to generate data which was used to estimate the unknown parameters of the model. Finally, the model and obtained parameters were found to be capable of reproducing previously observed effects of glutamine depletion on protein glycosylation. With further validation, this quantitative and mechanistic model could prove useful in aiding process development, control and optimisation for the manufacture of mAbs with desired glycosylation-associated quality.
